# Society and the Beggar

**Published:** 1895-03-16

**Authors:** 


### Social Problems.

### I.—SOCIETY AND THE BEGGAR.

A very remarkable series of investigations has been carried out during the last two or three years in Paris by M. Louis Paulian.* In Paris they do everything under a perfect system of organisation, even their begging, and, little by little, M. Paulian has succeeded in making his way behind the impenetrable barrier of secrecy which veils the craft, and has revealed to the astonished public the network of wires underlying their charity. The beggar, it would seem, can boast a far better organisation than the philanthropist. M. Paulian has tried both rôles, not once, but many times, and with unvarying results. As beggar, uniform success, every want supplied—food, lodging, clothing, pocket-money, railway tickets, medicine, baths, anything you please. "Mention any object whatever," he says, "an armchair, a violin, a pair of spectacles, a wooden leg, a baby's cradle, a bouquet of flowers, and immediately, under your very eyes, I will get you the offer of it from some charitable society." So much for the beggar.

As philanthropist, M. Paulian has nothing but a tale of failure to tell. The labour test, on which he principally relies, seems to be new to Paris, and unpopular even with the most destitute. It demands exceptional experience and discretion if it is to yield good results, but doubtless it was not in these qualities that M. Paulian and his confrères were wanting. Rather it would seem that it was the forces of the charitable themselves arrayed against him which lost him the battle. So many societies all aiming at the same result, all competing, as it were, for the beggar, have worked in Paris as in London his irretrievable downfall. He is insulted at the demand for a few hours' work in payment for the board and lodging he can get in twenty places for nothing, and sees no reason why he should prefer the low wage of unskilled labour to the rich premiums offered everywhere to idleness and imposture.

It must be thankfully recognised that many of the abuses still flourishing, according to M. Paulian, in Paris have been practically exterminated from the London streets. Street begging has been reduced to a minimum, and the most weakly generous have been educated to feel a twinge of shame at indiscriminate alms. The "exploiteurs" of infants and young children have met their match in Mr. Waugh. Even the medical charities are growing more wary, and the living would be poor and precarious indeed in this city which depended on the sale of wooden legs and surgical instruments obtained under various names.

The grand evil, however, arising from the want of harmonious inter-communication among different charitable agencies continues in England, as in France, to make the begging profession a profitable one. In view of the interesting attempt now being tried, under the presidency of the Lord Mayor, to discourage imposture and ensure the efficient relief of real distress, it may be useful to notice the system suggested by M. Paulian for the same purpose. The difference between the London and Parisian schemes is largely due to national characteristics. Voluntary effort is the essence of the Lord Mayor's scheme, and the aim is nothing short of harmonious co-operation among persons of the most widely differing religious opinions, acting in the areas best known to them. Centralisation is the main feature of M. Paulian's scheme; co-operation in any real sense he dismisses as a chimera, and replaces it by officialism, pure and simple. The various charitable agencies in despair of accord are to continue their conflicting methods; no attempt is to be made to unite them in aim and mutual understanding; but the *point d'accord* is to be a Central Bank (*caisse centrale*) entrusted with the duty of paying out all gifts, whether in money or kind, to every applicant. All donations, either from societies or private individuals, are to be made in the form of cheques payable by the Bank of Charities, which is charged solely with the duty of identifying the bearer, cashing his cheque, and recording the donation to his name. Should the list of gifts recorded mark a career of imposture, the bank is to be invested with the discretionary power of deferring payment until the donor can be consulted. For identification purposes the bank will furnish a card, to be produced on all future applications, inscribed with the applicant's anthropometric measurements, after the Bertillon system, with photograph attached. All this is very French. M. Paulian thinks no genuinely distressed person would object to having the length of his nose and the colour of his eyes inscribed against his name, but we fear this is a sanguine presumption. He is no doubt justified in urging that the registration of gifts paid to each applicant would prove an effectual barrier to misrepresentation and imposture, but that, after all, is only half the battle. The problem of how to give wisely still remains to be solved, and to render charity an act of pure officialism would tend not only seriously to lessen the responsibility of the giver, but also to efface from the gift its purest element of personal sympathy. An apparent gift, warranted to prove waste paper in the hands of the unworthy, seems rather an evasion than a solution of the difficulties of giving.

The system now on its trial in several districts in London, provides for the strictest investigation into the circumstances of each case, but leaves the ultimate relief of the applicant, now no longer a "case," to those most interested in his welfare. In this way only can the act of giving recover some of its old divine spontaneity, and the wise man's precept be obeyed in the wholesome exhortation, too often forgotten: "My son, blemish not thy good deeds; neither use uncomfortable words when thou givest anything. After thou hast given upbraid not."

* Paris qui Mendie. Mal et Remède, par Louis Paulian. (Paris: Paul Ollendorf.)

---

Fishermen in the sardine industry are subject to epidemics which take the form of whitlows on the fingers. They are said to be due to the handling of fish which are infected by certain species of bacteria. An interesting feature in the observation is that apparently two distinct species of bacteria are necessary for the production of these whitlows.

